# Inclusive Semiological Evaluation: Design and Validation of Protocols with an Intercultural and Sensory Approach

**DOI:** 10.1192/j.eurpsy.2026.11797

**Published:** 2026-07-31

**Authors:** Z. Miranda Sánchez, D. Sánchez-Pabón, K. Munera-Luque, A. Salinas-Miranda

**Affiliations:** Magdalena, Universidad Cooperativa de Colombia, Santa Marta, Colombia

## Abstract

**Introduction:**

Semiological evaluation in psychology requires more inclusive methodologies that ensure equitable access to relevant diagnostic processes for individuals with sensory disabilities or belonging to indigenous communities. This necessity calls for the development of adapted tools that consider functional, cultural, and linguistic diversity in clinical and educational contexts.

**Objectives:**

To design and validate inclusive semiological evaluation protocols that promote ethical, accessible, and culturally relevant clinical practice, integrating a differential approach into professional training.

**Methods:**

From an applied research perspective with a mixed-descriptive methodological design, protocols were developed through three phases: initial design of the instrument based on the characteristics of the target population; piloting with users from indigenous communities or with sensory disabilities; and a validation phase through direct user feedback and expert judgment from clinical psychology, inclusion, and assessment specialists. This final stage enabled fine-tuning to ensure the technical, linguistic, and cultural relevance of the instruments. The final products integrate both physical and digital adaptations, with visual, auditory, and linguistic accessibility criteria.

**Results:**

Since 2021, 80 interactive protocols have been developed by 267 students. These resources include versions in Braille, sign language, audio, readable fonts, visual contrasts, and availability in Spanish, English, Wayuunaiki, and Ika. They have been applied in clinical and educational contexts, and are currently used in university clinics, hospitals, and educational institutions

**Conclusions:**

This strategy strengthens methodological validation processes in semiological evaluation, promoting inclusion and equity in mental health. Its differential approach and practical application address SDGs 3, 4, 10, and 16, consolidating professional training committed to social justice.

**Disclosure of Interest:**

None Declared

